# Association between autophagy and inflammation in patients with rheumatoid arthritis receiving biologic therapy

**DOI:** 10.1186/s13075-018-1763-0

**Published:** 2018-12-05

**Authors:** Yi-Ming Chen, Chun-Yu Chang, Hsin-Hua Chen, Chia-Wei Hsieh, Kuo-Tung Tang, Meng-Chun Yang, Joung-Liang Lan, Der-Yuan Chen

**Affiliations:** 1 Division of Allergy, Immunology and Rheumatology, Department of Medical Research, Taichung Veterans General Hospital, Taichung City, Taiwan; 2 Faculty of Medicine, National Yang Ming University, Taipei, Taiwan; 3 Institute of Biomedical Science and Rong Hsing Research Center for Translational Medicine, Chung Hsing University, Taichung, Taiwan; 4 Rheumatology and Immunology Center, China Medical University Hospital, No. 2, Yude Road, Taichung, 40447 Taiwan; 5 Translational Medicine Laboratory, Rheumatic Diseases Research Center, China Medical University Hospital, Taichung, Taiwan; 6 School of Medicine, China Medical University, Taichung, Taiwan

**Keywords:** Autophagy, Inflammatory parameters, TNF-α inhibitors, Interleukin-6 receptor inhibitor, Rheumatoid arthritis (RA)

## Abstract

**Background:**

Increasing evidence indicates a pathogenic role of deregulated autophagy in rheumatoid arthritis (RA). We examined the relationship between autophagy and inflammatory parameters in patients with RA receiving biologic therapy.

**Methods:**

In 72 patients with RA and 20 healthy control subjects (HC), autophagosome levels were determined by the mean fluorescence intensity (MFI) of autophagosomotropic dye incorporated into circulating immune cells, and p62 expression levels in immune cells were measured by flow cytometry. We used immunoblotting to examine protein expression of LC3-II and p62 in peripheral blood mononuclear cells.

**Results:**

Patients with RA had significantly higher levels of autophagosome reflected by MFI of Cyto-ID in circulating lymphocytes, monocytes, and granulocytes (median values, 3.6, 11.6, and 64.8, respectively) compared with HC (1.9, 6.0, and 35.8; respectively) (all *p* < 0.001). p62 MFI levels in lymphocytes and granulocytes from patients with RA (17.1 and 8.6, respectively) were significantly lower than those in the corresponding cells from HC (20.2, *p* < 0.05; and 13.1, *p* < 0.001, respectively). Significantly higher levels of LC3-II protein expression in contrast to lower p62 protein levels were observed in patients with RA than in HC. The autophagosome levels in immune cells were significantly correlated with inflammatory parameters in patients with RA, and they were significantly decreased with disease remission after treatment with tumor necrosis factor-α inhibitors or interleukin-6 receptor inhibitor.

**Conclusion*s*:**

Elevated autophagy with significant correlation to inflammation suggests the involvement of autophagy in RA pathogenesis. The effectiveness of biologic therapy might be partly related to the downregulation of autophagy expression.

**Electronic supplementary material:**

The online version of this article (10.1186/s13075-018-1763-0) contains supplementary material, which is available to authorized users.

## Background

Autophagy is the process of engulfment and degradation of cytoplasmic contents by lysosomes [1, 2]. Autophagy initiation is regulated by the Unc51-like kinase 1 complex [3], and the most critical step in autophagy is autophagosome formation through the conjugation of microtubule-associated protein light chain 3 (LC3) with phosphatidylethanolamine [2]. LC3 consists of a soluble form (LC3-I, 18 kDa) and a lipidated form (LC3-II, 16 kDa). The LC3-binding adaptor p62 (sequestosome 1 [SQSTM1]) binds ubiquitinated substrates, serves as a bridge for the delivery to autophagosome, and then promotes their degradation through a proteasomal pathway [4, 5]. Therefore, decreased p62 levels are associated with autophagy activation. Finally, the autophagosome fuses with a lysosome to form an autolysosome that digests the engulfed cargo [1, 2].

The networks formed by autophagy and inflammation are complex. Autophagy is involved in the induction and suppression of inflammation and vice versa [6–8]. Proinflammatory cytokines such as tumor necrosis factor (TNF)-α and interleukin (IL)-6 have been shown to stimulate autophagy, and autophagy also contributes to the secretion of these cytokines [9–12]. However, autophagy is tightly regulated in its response to inflammation, such as participating in the clearance of protein complexes (e.g., inflammasomes) through proteasomal degradation [13]. Owing to the multifaceted roles of autophagy in inflammatory responses [6–8], deregulated autophagy has been implicated in the pathogenesis of autoimmune diseases [12, 14, 15].

Rheumatoid arthritis (RA) is an inflammatory disease that leads to chronic synovitis and joint erosion [16]. Proinflammatory cytokines such as TNF-α and IL-6 can promote synovitis, cartilage damage, and bone destruction [16–18]. The importance of TNF-α and IL-6 in RA pathogenesis is supported by the therapeutic effectiveness of biologics targeting cytokines [18–20]. With autophagy tightly regulated to ensure immune homeostasis, its deregulation may serve a pathogenic role in RA [12, 21, 22]. Lin et al. demonstrated that autophagy is activated in RA in a TNF-α-dependent manner in murine model [21], and Connor et al. revealed that TNF-α stimulated autophagy through the induction of endoplasmic reticulum stress response [22]. Recent studies also indicated an increased autophagy in RA fibroblast-like synoviocytes (FLS) but not in osteoarthritis FLS [12, 23, 24], with a dual role of autophagy in regulating the death pathway in RA FLS [25]. In spite of the accumulating evidence supporting the critical role of autophagy in RA [23–25], there were still limited data regarding the association of autophagy with inflammation in human RA.

In this pilot study, we compared the difference in autophagy expression between patients with RA and healthy control subjects (HC). We also examined the correlation between autophagy expression and inflammatory parameters in patients with RA. In addition, we examined the changes of autophagy expression and serum cytokine levels in patients with RA after 6-month therapy with biologics or conventional synthetic disease-modifying antirheumatic drugs (csDMARDs) alone.

## Methods

### Subjects

In this prospective study, 72 patients with RA who fulfilled the 2010 classification criteria of the American College of Rheumatology/European League Against Rheumatism collaborative initiative [26] were consecutively enrolled. Disease activity was assessed using the 28-joint Disease Activity Score (DAS28) [27], with active status defined as a DAS28 score > 3.2 [28]. Sixty biologic-naïve patients with active RA who had received csDMARDs started therapy with TNF-α inhibitors (etanercept or adalimumab, *n* = 28) or IL-6R inhibitor (tocilizumab, *n* = 32) in combination with a stable weekly dose of methotrexate 7.5–15 mg according to the guidelines [29], and the other 12 patients continued with csDMARD therapy alone. Twenty age- and sex-matched healthy volunteers served as HC. The Institutional Review Board of Taichung Veterans General Hospital approved this study (CE14307B), and written consent was obtained from each participant according to the Declaration of Helsinki.

### Quantitation of autophagosome levels in circulating immune cells by Cyto-ID staining

Cyto-ID, a cationic amphiphilic tracer dye, specifically recognizes autophago(lyso)some and can be quantified using flow cytometry [30]. To determine autophagosome levels in circulating immune cells, the fluorescence of Cyto-ID on the cells was measured by using the Cyto-ID™ Autophagy Detection Kit (Enzo Life Sciences, Farmingdale, NY, USA) according to the manufacturer’s protocol and the described technique [31–33]. Briefly, 100 μl of whole blood was stained with 0.25 μl/ml of Cyto-ID Green Autophagy Detection Reagent (Enzo Life Sciences) and 20 μl of phycoerythrin-cyanine 5 (PC5)-conjugated CD45-specific monoclonal antibody (mAb) (Beckman Coulter Life Sciences, Indianapolis IN, USA). Incubation with CD3-, CD14-, CD66b-, and CD45-specific antibodies was done simultaneously with autophagy dye. After incubation for 30 min in the dark at room temperature (RT), cells were reacted with OptiLyse Solution (Beckman Coulter Life Sciences) for 10 min to lyse red blood cells. After PBS washing, cells were analyzed by flow cytometry (Beckman Coulter Life Sciences). Monocytes, lymphocytes, and granulocytes were gated on the basis of CD45^+^ side scatter, and at least 1 × 10^4^ cells from each sample were analyzed. To verify the gated lymphocytes, monocytes, and granulocytes, 100-μl blood samples were stained with 20 μl of fluorescein isothiocyanate (FITC)-conjugated CD3-specific mAb (Beckman Coulter Life Sciences), 20 μl of PC5-conjugated CD14-specific mAb, and 20 μl of FITC-conjugated CD66b-specific mAb, respectively, with 20 μl of PC5-conjugated CD45-specific mAb separately for 15 min at RT. Regarding the subgroups of lymphocytes, 100-μl blood samples were stained with 5 μl of FITC-conjugated CD4-specific mAb (BioLegend, San Diego, CA, USA), 5 μl of PC5-conjugated CD8-specific mAb (Beckman Coulter Life Sciences), and 10 μl of PC5-conjugated CD19-specific mAb (Beckman Coulter Life Sciences) with 5 μl of peridinin chlorophyll protein (PerCP)-conjugated CD3-specific mAb (BD, Franklin Lakes, NJ, USA) separately with autophagy dye for 30 min at RT. Data were expressed as the mean fluorescence intensity (MFI) of Cyto-ID staining. We also examined autophagosome levels, determined by Cyto-ID staining, in both synovial fluid (SF)-derived and peripheral blood (PB)-derived immune cells from two patients with active RA.

### Quantitation of autophagic adaptor p62 levels in immune cells using flow cytometry

Intracellular immunofluorescent staining of p62 molecule was performed following fixation and permeabilization using the modified method of a previous study [33]. Briefly, 50 μl of whole blood was stained with 20 μl of FITC-conjugated CD45-specific mAb for 15 min at RT. Cells were fixed by adding 100 μl of reagent 1 (Beckman Coulter Life Sciences) for 15 min and were centrifuged for 5 min at 300 × *g*. After removal of the supernatant, 100 μl of reagent 2 (Beckman Coulter Life Sciences) was added for permeabilization for 10 min, and cells were subsequently incubated with PerCP-conjugated p62/SQSTM1 mAb (clone 5H7E2; Novus Biologicals, Littleton, CO, USA) for 15 min in the dark at RT. PerCP-conjugated immunoglobulin G1 (R&D Systems, Minneapolis, MN, USA) was used as an isotype control. Cells were immediately analyzed using flow cytometry (Beckman Coulter Life Sciences).

### Determination of autophagy expression using Western blot analysis

Total proteins were extracted from peripheral blood mononuclear cell (PBMC) lysates from 25 patients with active RA and 10 HC. The proteins were separated by 10–12% SDS-PAGE and then transferred to polyvinylidene fluoride membranes (Bio-Rad Laboratories, Hercules, CA, USA). Immunoblots were performed using primary antibodies (1:1000 dilution) overnight at 4 °C against LC3-II (Abcam, Cambridge, MA, USA), p62 (Abcam), and glyceraldehyde 3-phosphate dehydrogenase (GAPDH) (Santa Cruz Biotechnology, Dallas, TX, USA), followed by incubation with horseradish peroxidase-conjugated antirabbit secondary antibody (1:5000) for 1 h at 37 °C (Santa Cruz Biotechnology). The luminescent signal was detected by using the Fujifilm LAS-3000 image detection system (Fujifilm, Tokyo, Japan), and image processing and data quantification were performed using Multi Gauge version 2.02 software (Fujifilm). The LC3-II/LC3-I ratio was calculated as LC3-II expression levels divided by LC3-I expression levels, and the p62 expression levels were normalized to GAPDH.

### Plasma antioxidant capacity

The measurement of the total antioxidant capacity (TAC) of biological fluids provides an indication of the overall capability to counteract ROS. Plasma levels of TAC were measured using a colorimetric assay kit (BioVision Incorporated, Milpitas, CA, USA). 6-Hydroxy-2,5,7,8-tetramethylchroman-2-carboxylic acid (Trolox) was used to standardize antioxidants, with all the other antioxidants being measured in Trolox equivalents. The Cu^2+^ was reduced to Cu^+^ by the antioxidant factors in the sample coupled with a colorimetric probe. For calibration, 1 mM Trolox in dimethyl sulfoxide-water was used. Each microtiter plate was filled with either 100 μl of calibrators (0, 4, 8, 16, or 20 nmol Trolox) or 100 μl of diluted serum. Then, 100 μl of freshly prepared Cu^2+^ working solution was added, and the mixture was incubated at RT for 1.5 h. The sample absorbance was analyzed at 570 nm as a function of Trolox equivalent concentrations according to the manufacturer’s instructions. The antioxidant capacity was presented in nmol/μl.

### Determination of serum levels of proinflammatory cytokines

Serum levels of TNF-α and IL-6 were determined using an enzyme-linked immunosorbent assay (PeproTech Inc., Rocky Hill, NJ, USA) according to the manufacturer’s instructions.

### Statistical analysis

Results are presented as the mean ± SD or median (IQR). The Mann-Whitney *U* test was used for between-group comparison of autophagy expression, cytokine levels, and oxidative stress status evidenced by TAC levels. The correlation coefficient was obtained by Spearman’s rank test. For evaluation of the changes of autophagy expression and serum cytokine levels during the follow-up period in patients with RA, the Wilcoxon signed-rank test was employed. *p* < 0.05 was considered significant.

## Results

### Clinical characteristics of patients with RA

As illustrated in Table 1, 69.4% of patients with RA were positive for rheumatoid factor (RF), and 61.1% were positive for anticitrullinated peptide antibodies (ACPA). As expected, patients with RA scheduled for biologic therapy had higher disease activity at baseline than those receiving csDMARDs alone. However, there were no significant differences in the positive rates of RF or ACPA, daily dose of corticosteroids, or the proportion of used csDMARDs among patients with RA receiving different therapies. There were no significant differences in demographic data between patients with RA and HC.

**Table 1:** Clinical characteristics, laboratory findings, and autophagy expression at baseline

	TNF-α inhibitors (*n* = 28)	IL-6R inhibitor (*n* = 32)	csDMARDs alone (*n* = 12)	HC (*n* = 20)
Mean age (years)	56.7 ± 12.1	55.5 ± 14.1	58.1 ± 14.3	53.3 ± 11.4
Female (%)	22 (78.6%)	24 (75.0%)	10 (83.3%)	15 (75.0%)
RF positivity (%)	22 (78.6%)	20 (62.5%)	8 (66.7%)	NA
ACPA positivity (%)	19 (67.9%)	18 (56.3%)	7 (58.3%)	NA
ESR (mm/first hour)	44.4 ± 30.0*	35.8 ± 21.8	30.5 ± 22.6	NA
CRP (mg/dl)	2.3 ± 2.5*	2.0 ± 2.3	1.3 ± 1.4	NA
DAS28 at baseline	5.89 ± 0.59*	5.95 ± 0.70*	4.80 ± 0.71	NA
Daily steroid dose (mg)	6.3 ± 1.6	6.5 ± 1.5	5.6 ± 1.9	NA
Baseline csDMARDs				
MTX + SSZ + HCQ	23 (82.1%)	26 (81.3%)	10 (83.4%)	NA
SSZ + HCQ + Cyc	2 (7.1%)	3 (9.4%)	1 (8.3%)	NA
MTX + SSZ	1 (3.6%)	1 (3.1%)	0 (0.0%)	NA
MTX + SSZ + Cyc	1 (3.6%)	0 (0.0%)	1 (8.3%)	NA
MTX+ SSZ + HCQ + Cyc	1 (3.6%)	2 (6.2%)	0 (0.0%)	NA

*Abbreviations: ACPA* Anticitrullinated peptide antibodies, *CRP* C-reactive protein, *csDMARDs* Conventional synthetic disease-modifying antirheumatic drugs, *Cyc* Cyclosporine, *DAS28* Disease Activity Score in 28 joints, *ESR* Erythrocyte sedimentation rate, *HCQ* Hydroxychloroquine, *IL-6R* Interleukin-6 receptor, *MTX* Methotrexate, *NA* Not applicable, *RF* Rheumatoid factor, *SSZ* Sulfasalazine, *TNF-α* Tumor necrosis factor-α

Data are presented as mean ± SD, number (percent), or median (25th–75th quartiles)

*p < 0.05 vs. HC by Mann-Whitney *U* test for between-group comparison of numerical variables

### MFI of Cyto-ID in circulating immune cells from patients with RA and HC

**Fig. 1:**
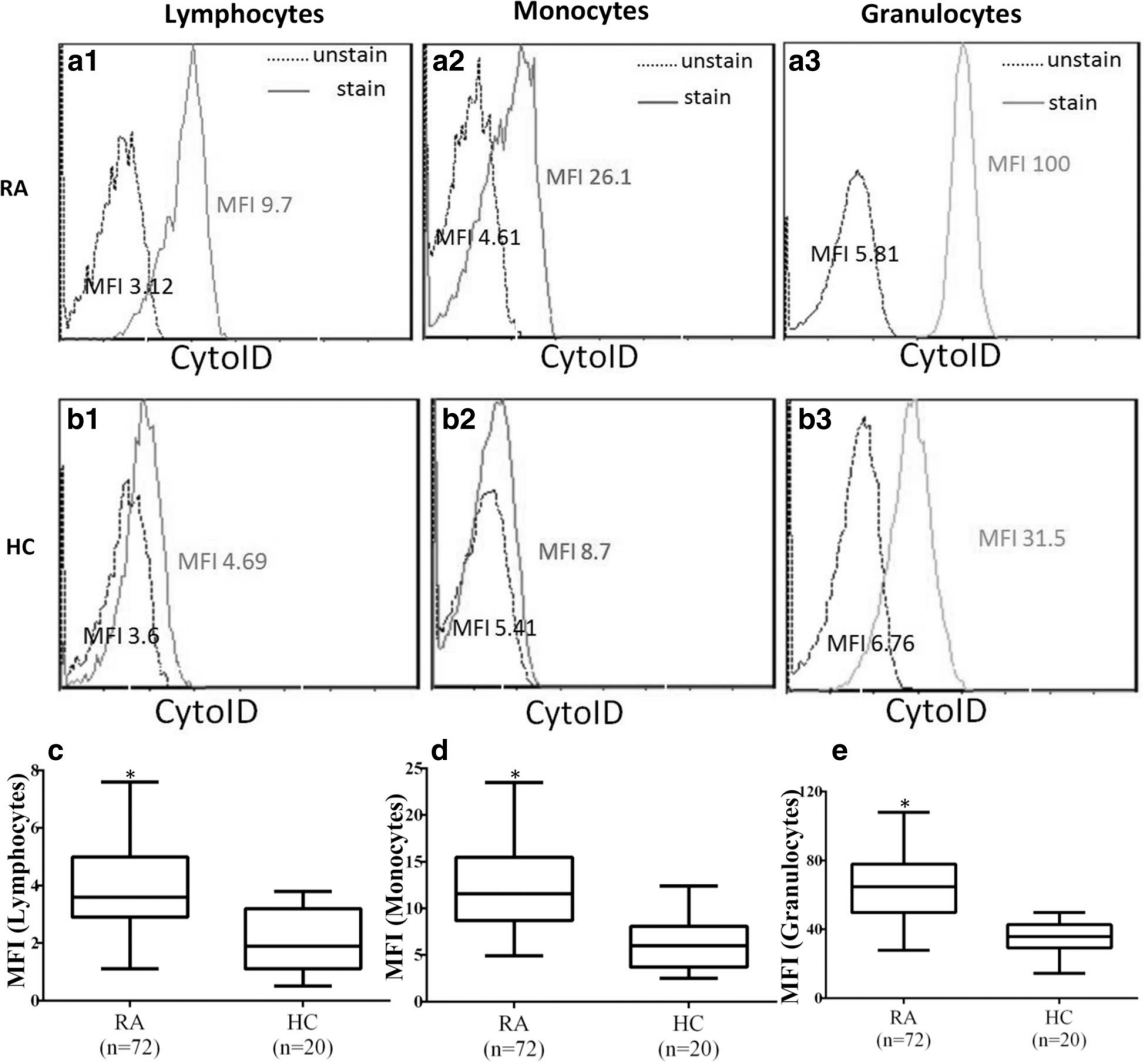
Representative cytometric histograms of Cyto-ID staining in lymphocytes (**a**1 and **b**1), monocytes (**a**2 and **b**2), and granulocytes (**a**3 and **b**3) from one patient with rheumatoid arthritis (RA) and one healthy control subject (HC). Comparisons of autophagosome levels reflected by Cyto-ID staining mean fluorescence intensity in lymphocytes (**c**), monocytes (**d**), and granulocytes (**e**) between patients with RA and HC. Data are presented as box plot diagrams, with the box encompassing the 25th percentile (lower bar) to the 75th percentile (upper bar). The horizontal line within the box indicates the median value for each group. *p < 0.001 vs. HC

Representative cytometric histograms of Cyto-ID-staining obtained from one patient with RA and one HC are shown in Fig. 1a and b. Significantly higher values of MFI were observed in circulating lymphocytes, monocytes, and granulocytes from patients with RA (median 3.6, IQR 2.9–5.0; 11.6, IQR 8.7–15.5; 64.8, IQR 49.1–78.1; respectively) compared with those from HCs (1.9, IQR 1.1–3.2; 6.0, IQR 3.7–8.1; 35.8, IQR 29.3–42.7; respectively, all *p* < 0.001) (Fig. 1c–e).

### MFI of Cyto-ID in circulating CD4^+^ T cells, CD8^+^ T cells, and CD19^+^ B cells from patients with RA and HC

Representative cytometric histograms of MFI of Cyto-ID in circulating CD4^+^ T cells, CD8^+^ T cells, and CD19^+^ B cells obtained from one patient with RA and one HC are shown in Additional file 1: Figure S1A. Significantly higher values of MFI were observed in circulating CD4^+^ and CD8^+^ T cells from patients with RA (median 93.5, IQR 69.4–126.3; 114.0, IQR 87.5–143.0, respectively) than in those from HC (median 46.7, IQR 25.5–71.0; 54.0, IQR 30.5–82.8, both *p* < 0.05) (Fig. 1b, c). However, no significant difference in MFI of CD19^+^ B cells was observed between patients with RA and HC (median 439.5, IQR 205.0–1187.5 versus 500, IQR 135.5–750.7) (Fig. 1d).

### MFI of Cyto-ID in synovial fluid immune cells from patients with RA

Given that immune cells barely exist in SF of patients with osteoarthritis or HC, their PB-derived immune cells were used to serve as controls. Our results showed that MFI of Cyto-ID was significantly higher in SF granulocytes (7846 and 8857, respectively) than in PB-derived granulocytes (2131 and 2364, respectively) from two patients with active RA (Additional file 1: Figure S1E–G).

### MFI of p62 in circulating immune cells from patients with RA and HC

**Fig. 2:**
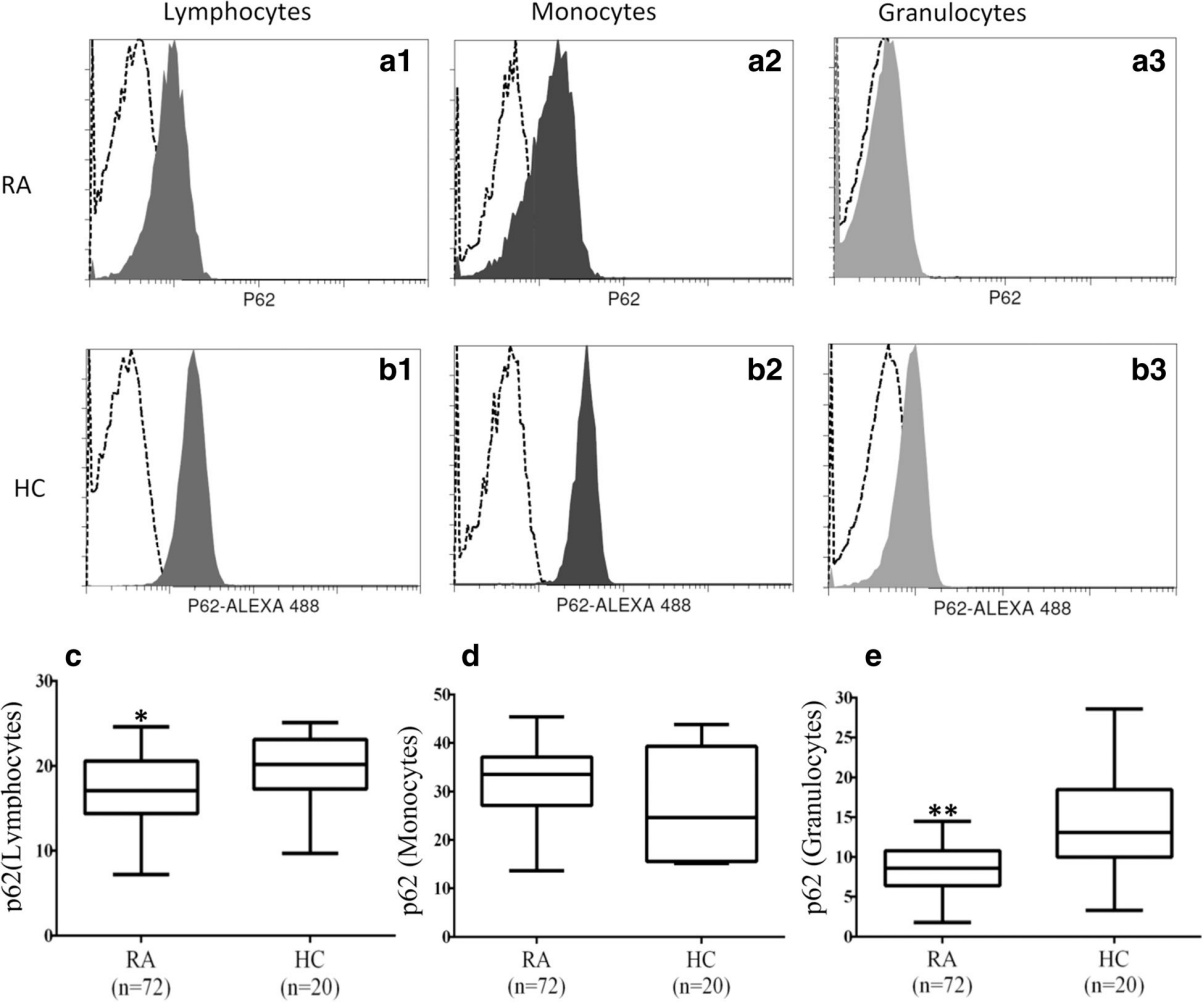
Representative cytometric histograms of p62 levels in lymphocytes (**a**1 and **b**1), monocytes (**a**2 and **b**2), and granulocytes (**a**3 and **b**3) from one patient with rheumatoid arthritis (RA) and one healthy control subject (HC). Gray shadows indicate cytometric histograms of the stained p62 expression in immune cells from patients with RA and HC. Comparisons of p62 mean fluorescence intensity in lymphocytes (**c**), monocytes (**d**), and granulocytes (**e**) between patients with RA and HC. Data are presented as box plot diagrams, with the box encompassing the 25th percentile (lower bar) to the 75th percentile (upper bar). The horizontal line within the box indicates the median value for each group. *p < 0.05, **p < 0.001 vs. HC

Representative examples of cytometric histograms of p62 levels obtained from one patient with active RA and one HC are shown in Fig. 2a and b. Significantly lower MFI values of p62 were observed in circulating lymphocytes and granulocytes from patients with RA (median 17.1, IQR 14.4–20.6; and 8.6, IQR 6.4–10.8, respectively) than in those from HC (20.2, IQR 17.3–23.1, *p* < 0.05; and 13.1, IQR 10.0–18.5, *p* < 0.001; respectively) (Fig. 2c and e). However, there was no difference in the MFI of p62 in circulating monocytes between patients with RA and HC.

### Plasma levels of total antioxidant capacity in patients with RA and HC

Significantly lower TAC levels (median 55.1 nmol/μl, IQR 50.3–61.5 nmol/μl) were shown in patients with RA than in HC (59.0 nmol/μl, IQR 56.2–65.8 nmol/μl; *p* < 0.05). Plasma TAC levels were also negatively associated with autophagosome levels reflected by Cyto-ID MFI in circulating granulocytes from patients with RA (*r* = − 0.313, *p* < 0.01).

### Serum cytokine levels in patients with RA and HC

Patients with RA had significantly higher levels of TNF-α and IL-6 (median 172 pg/ml, IQR 117–308 pg/ml; and 917 pg/ml, IQR 455–2842 pg/ml, respectively) compared with HC (82 pg/ml, IQR 50–97 pg/ml; and 231 pg/ml, IQR 159-452 pg/ml, respectively; *p* < 0.001).

### Correlations between autophagy expression and inflammatory parameters in RA

As illustrated in Table 2, DAS28 and C-reactive protein (CRP) levels at baseline were significantly and positively correlated with the autophagosome levels in circulating lymphocytes, monocytes, and granulocytes. Serum TNF-α levels were also positively correlated with the autophagosome levels in lymphocytes, monocytes, and granulocytes, and serum IL-6 levels were positively correlated with the autophagosome levels in lymphocytes. On the contrary, DAS28 scores were negatively correlated with p62 levels in the circulating lymphocytes, monocytes, or granulocytes, and CRP levels were negatively with p62 levels in lymphocytes. Serum TNF-α levels were also negatively correlated with p62 levels in lymphocytes, monocytes, and granulocytes. However, there was no significant association between the levels of autophagosome or p62 and ACPA levels in patients with RA (data not shown).

**Table 2:** Correlation between autophagy expression and inflammatory parameters in 72 patients with rheumatoid arthritis

Autophagy expression	DAS28	CRP	TNF-α	IL-6
Cyto-ID MFI in lymphocyte	0.522***	0.285*	0.325**	0.252*
Cyto-ID MFI in monocyte	0.478***	0.262*	0.320**	0.200
Cyto-ID MFI in granulocyte	0.486***	0.365**	0.247*	0.141
P62 MFI in lymphocyte	−0.309**	−0.297*	−0.336**	− 0.197
P62 MFI in monocyte	−0.325**	−0.221	− 0.293*	−0.188
P62 MFI in granulocyte	−0.249*	−0.130	− 0.258*	−0.022

*Abbreviations: CRP* C-reactive protein, *DAS28* Disease Activity Score in 28 joints, *IL-6* Interleukin 6, *MFI* Mean fluorescence intensity, *TNF-α* Tumor necrosis factor-α

*p < 0.05, **p < 0.01, ***p < 0.001 were determined by Spearman’s rank-correlation test

### Autophagy protein expression in PBMCs from patients with RA and HC

**Fig. 3:**
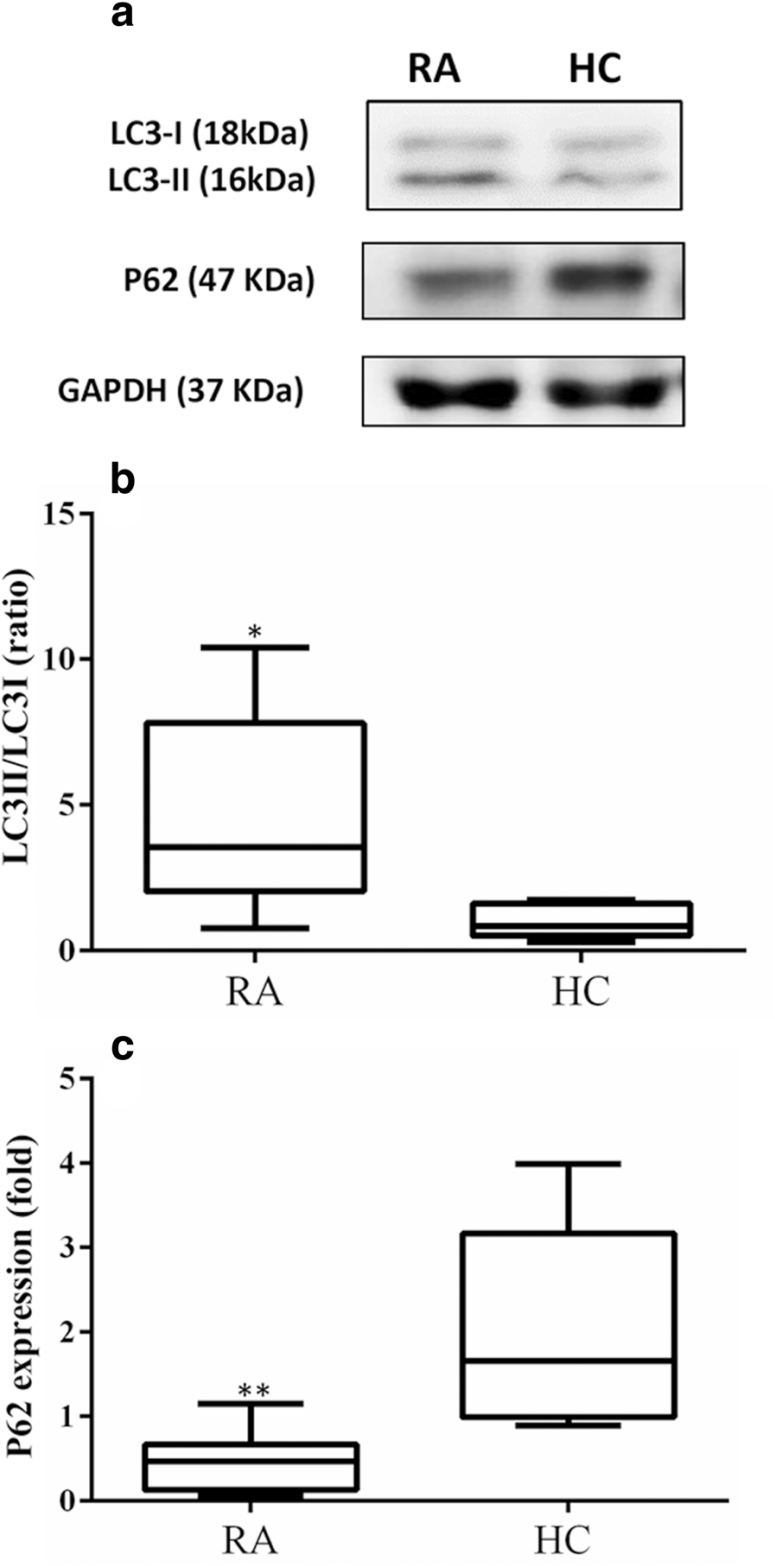
Representative example of LC3-II/LC3-I and p62 protein expression in peripheral blood mononuclear cell lysates from one patient with rheumatoid arthritis (RA) and one healthy control subject (HC) (**a**). Comparisons of protein expression levels of LC3-II/LC3-I (**b**) and p62 (**c**) in patients with RA and HC are shown. Data are presented as box plot diagrams, with the box encompassing the 25th percentile (lower bar) to the 75th percentile (upper bar). The horizontal line within the box indicates median value for each group. *p < 0.005, **p < 0.001 versus HC, determined by Mann-Whitney *U* test

Representative immunoblot analyses of autophagy expression in PBMC lysates were obtained from one patient with active RA and one HC (Fig. 3a). The LC3-II expression levels were significantly higher in patients with active RA (median 3.55, IQR 2.05–7.82) than in HC (0.86, IQR 0.52–1.63; *p* < 0.005) (Fig. 3b). In contrast, patients with RA had significantly lower levels of p62 expression (0.47, IQR 0.13–0.67) than HC (1.66, IQR 0.99–3.17; *p* < 0.001) (Fig. 3c).

Given that lymphocytes and monocytes comprise the majority of PBMCs, we estimated autophagosome levels in PBMCs by summing the Cyto-ID MFI in both lymphocytes and monocytes. We examined the correlation between autophagy protein expression and autophagosome levels in PBMCs. The results showed a positive correlation between LC3-II expression levels and autophagosome levels (*r* = 0.573, *p* < 0.01) and a negative correlation between p62 levels in immunoblotting and autophagosome levels in Cyto-ID staining (*r* = − 0.423, *p* < 0.05).

### Change of autophagy expression and serum cytokine levels in patients with RA after 6 months of therapy

**Fig. 4:**
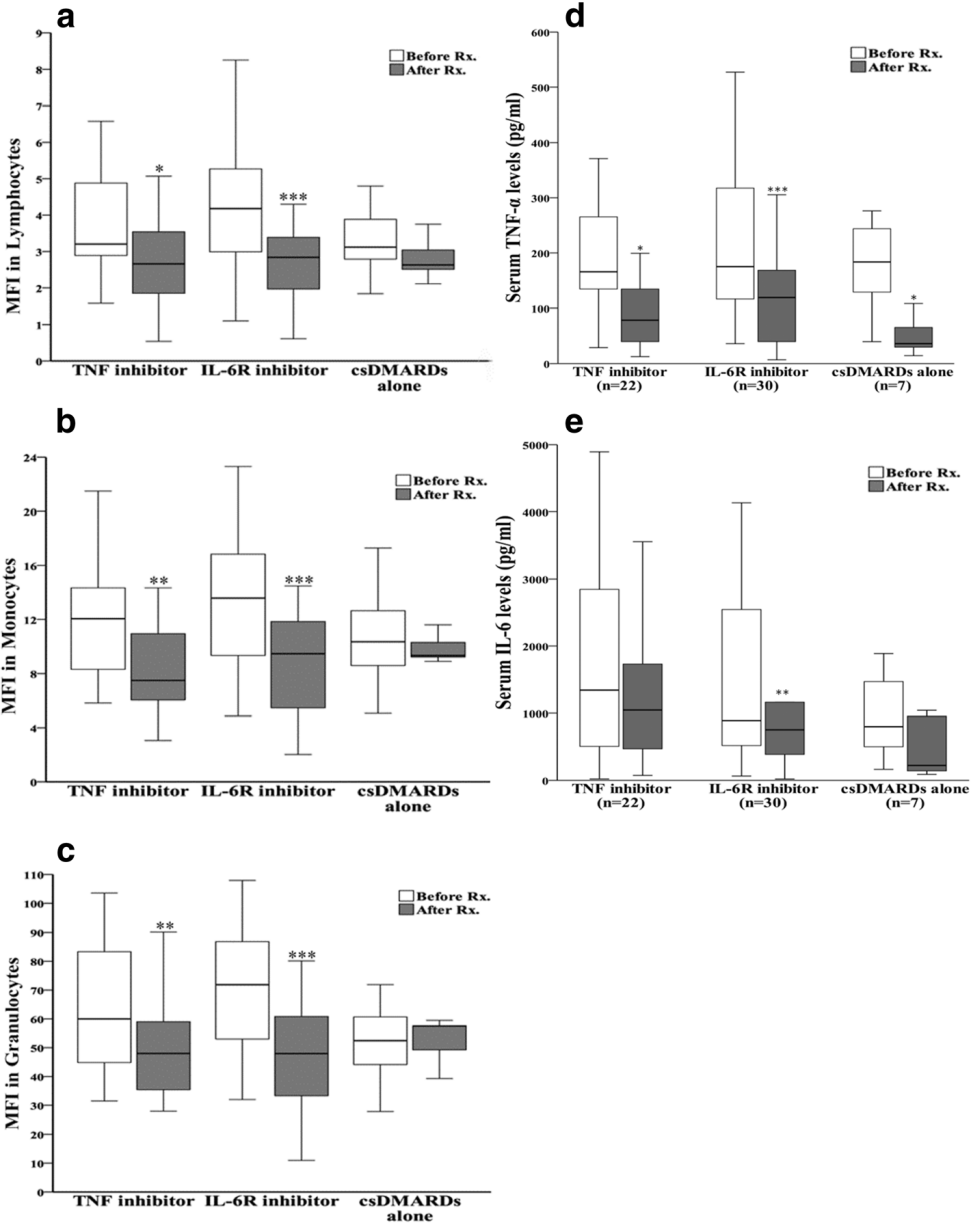
The changes in autophagosome levels evidenced by Cyto-ID mean fluorescence intensity in circulating (**a**) lymphocytes, **b** monocytes, and (**c**) granulocytes and the change in (**d**) serum tumor necrosis factor-α levels as well as (**e**) interleukin (IL)-6 levels after 6-month therapy in patients with rheumatoid arthritis. Data are presented as the mean ± SEM. *p < 0.05, **p < 0.005, ***p < 0.001 vs. before treatment, as determined by Wilcoxon signed-rank test

Sixty patients were available for examining autophagy expression before (at baseline) and after 6-month biologic therapy or csDMARDs alone. As shown in Fig. 4a, the autophagosome levels of circulating lymphocytes, monocytes, and granulocytes significantly declined (median 3.2, IQR 2.8–4.9 vs. 2.7, IQR 1.6–3.8, *p* < 0.05; 12.1, IQR 8.2–15.2 vs. 7.5, IQR 5.8–11.0, *p* < 0.005; 60.0, IQR 44.7–86.0 vs. 48.0, IQR 34.7–61.0, *p* < 0.005; respectively), paralleling the decrease of DAS28 (6.0, IQR 5.4–6.4 vs. 3.9, IQR 3.2–4.5, *p* < 0.001) in patients after 6-month anti-TNF-α therapy. In patients with RA receiving different TNF-α inhibitors, there was no significant difference in the change of autophagy expression between etanercept-treated and adalimumab-treated patients.

In patients after 6-month anti-IL-6R therapy (Fig. 4b), MFI of Cyto-ID in lymphocytes, monocytes, and granulocytes significantly declined (4.2, IQR 3.0–5.3 vs. 2.8, IQR 1.9–3.8; 13.5, IQR 9.3–16.8 vs. 9.5, IQR 5.5–11.9; 71.3, IQR 53.0–86.8 vs. 49.2, IQR 33.3–61.1, all *p* < 0.001), paralleling the decrease of DAS28 (6.0, IQR 5.4–6.5 vs. 3.2, IQR 3.0–3.8, *p* < 0.001). Although DAS28 also significantly decreased (5.2, IQR 4.2–5.9 vs. 3.1, IQR 3.0–3.9, *p* < 0.05) in those receiving csDMARDs alone, there was no significant change in MFI values of Cyto-ID in circulating lymphocytes, monocytes, or granulocytes (Fig. 4c).

Regarding the changes in serum cytokine levels, TNF-α levels significantly declined in patients with RA receiving any of the following medications for 6 months: TNF-α inhibitor, IL-6R inhibitor, or csDMARDs alone (median 165.8 pg/ml, IQR 132.8–265.4 pg/ml vs. 78.5 pg/ml, IQR 38.8–136.0 pg/ml, *p* < 0.01; 175.2 pg/ml, IQR 114.0–324.3 pg/ml vs. 119.2 pg/ml, IQR 39.7–168.6 pg/ml, *p* < 0.001; 183.6 pg/ml, IQR 90.9–276.3 pg/ml vs. 36.1 pg/ml, IQR 25.2–93.1, *p* < 0.05). Although serum IL-6 levels also decreased significantly (873.9 pg/ml, IQR 470.2–2545.1 pg/ml vs. 752.9 pg/ml, IQR 373.9–1163.0 pg/ml, *p* < 0.005) in patients with RA receiving IL-6R inhibitor, serum IL-6 levels did not show significant changes in those receiving TNF-α inhibitors (median 1342.3 pg/ml, IQR 462.5–2869.7 pg/ml vs. 1044.8 pg/ml, IQR 428.4–1801.1 pg/ml, *p* = 0.277) or csDMARDs alone (median 799.9 pg/ml, IQR 449.2–1887.9 pg/ml vs. 223.3 pg/ml, IQR 121.0–1042.3 pg/ml, *p* = 0.128) (Fig. 4d, e).

## Discussion

Although autophagy has recently emerged as an important regulator in the induction and maintenance of joint inflammation [8, 21–25, 34], the pathogenic association between autophagy and inflammation in RA has rarely been explored. To our knowledge, the present study is the first to demonstrate significantly higher levels of autophagosomes, as evidenced by the MFI of Cyto-ID, in circulating immune cells from patients with RA than in HC. The protein expression of LC3-II, an indicator of autophagosome formation, was also elevated in patients with RA. We also revealed significantly lower p62 levels in patients with RA than in HC, as revealed by flow cytometry and immunoblot analyses, indicating increased autophagic activity in patients with RA. In addition, the autophagosome levels in circulating immune cells were significantly correlated with inflammatory parameters in patients with RA. These observations suggest a potential involvement of activated autophagy in RA pathogenesis.

Autophagosome formation, a critical step in the autophagic process [1, 4], was significantly increased in circulating lymphocytes, monocytes, and granulocytes from our patients with RA. Among the circulating lymphocytes, significantly higher levels of autophagosome were observed in circulating CD4^+^ T and CD8^+^ T cells, but not in CD19^+^ B cells, from patients with RA than in HC. Van Loosdregt et al. similarly found an increased autophagy in CD4^+^ T cells and CD8^+^ T cells of patients with RA compared with HC [34]. We also revealed that the elevated protein expression of LC3-II, an indicator of autophagosome formation [1, 2], was positively associated with Cyto-ID MFI in circulating immune cells from patients with RA. Moreover, autophagosome levels were markedly higher in SF granulocytes than in PB-derived granulocytes in patients with active RA (Additional file 1: Figure S1F–H), in agreement with other recent reports showing that autophagy expression is higher in granulocytes from SF than in those from PB in patients with RA [35]. Previous reports also showed an increased autophagy in RA and persistent activation of the autophagy pathway in FLS from patients with RA or in murine arthritis [23–25]. Therefore, it is reasonable to speculate that increased autophagy in immune cells from patients with RA may result in their persistent activation, particularly at the site of inflammation. In our study, autophagosome levels in immune cells from patients with RA were positively correlated with DAS28, CRP levels, and TNF-α values. Moreover, the autophagosome levels in circulating immune cells declined significantly, paralleling the decrease of serum TNF-α values, in our patients with RA undergoing effective treatment. These observations suggest an association between elevated autophagy and RA-related inflammation, as has been shown in other previous studies [21, 36].

Excessive generation of ROS driven by overproduction of proinflammatory cytokines such as TNF-α participates in an inflammatory process in RA. An efficient antioxidant system catalyzes the inactivation of ROS. Previous studies have revealed increased oxidative stress along with low antioxidant levels and reduced antioxidant capacity in plasma of patients with RA [37]. Plasma TAC levels, as determined in our study, reflect the global combined antioxidant capacity of all individual antioxidants in plasma. We have demonstrated significantly lower TAC levels in patients with RA than in HC, indicating an increased oxidative stress in human RA [37]. Zhang et al. also revealed that excessive ROS cause mitochondrial damage and then induce autophagy in adjuvant-arthritis (AA) rats [38]. In agreement with their findings that resveratrol, an antioxidant, could suppress oxidative stress by reducing autophagy expression in AA rats [38], an inverse correlation between plasma TAC levels and autophagosome levels in circulating granulocytes was observed in our patients with RA.

With p62-bound ubiquitinated substrates incorporated into the autophagosome and then degraded into the autolysosomes, p62 level is selectively degraded by autophagy [39] and serves as a readout of autophagic flux [4, 5]. Thus, the decreases of p62 levels reflect autophagic activation [40]. In the present study, the p62 MFI in circulating immune cells and p62 protein expression in PBMCs from patients with RA were significantly lower than in those from HC, with the p62 MFI in immune cells negatively correlated with RA inflammatory parameters (Table 2). The combination of increased autophagosome formation and decreased p62 levels suggests autophagic activation in RA. Yang et al. demonstrated the upregulated expression of LC3-II as well as decreased p62 expression in RA FLS [12], also indicating an activated autophagy in RA.

Consistent with previous reports [16–18], significantly higher levels of serum inflammatory cytokines, including TNF-α and IL-6, were found in our patients with RA than in HC. The positive correlation between TNF-α levels and autophagosome levels in circulating immune cells from patients with RA further supports the findings that TNF-α could stimulate the conversion of LC3-I into LC3-II, an indicator of autophagosome formation [21]. In addition, we revealed a significant reduction of autophagosome levels, serum TNF-α levels, and disease activity in patients with RA after 6-month anti-TNF-α therapy (Fig. 4). In an animal model of RA, the inhibition of autophagy could also alleviate synovial inflammation [41]. The inhibitory effect of anti-TNF-α therapy on autophagy may be responsible for its associated increase of infection risk, particularly tuberculosis [10]. Besides, the positive correlation between serum IL-6 levels and autophagosome levels in circulating lymphocytes is in agreement with the previous finding that the knockdown of autophagic initiation ameliorates activated lymphocyte-derived DNA-induced murine lupus through an inhibition of IL-6 [42]. The significant reduction of autophagy expression in our patients with RA after 6-month anti-IL-6R therapy is also similar to previous reports that anti-IL6R therapy was effective in the treatment of glioblastoma by blocking autophagy [43], and the inhibition of autophagy could reduce osteoclastogenesis and prevent structural damage in RA [21]. However, whether the changes in autophagy expression following biologic therapy are related to a cytokine blocking effect needs to be further validated.

In spite of the novel findings in this pilot study, there were still some limitations. We enrolled a limited number of patients with active RA who were followed throughout 6-month therapies. Because the medications used, such as corticosteroids, may influence autophagy through downregulating proinflammatory cytokine secretion [44], their interference should be considered. In contrast to the results of a previous report in an early RA cohort [45], we did not reveal a significant association between autophagy expression and ACPA levels in patients with RA. This discrepancy may be explained by the fact that most of the patients enrolled in our study were not in an early RA stage. Therefore, a long-term study enrolling a larger group of patients, including an early RA population, is required for the validation of our findings.

Last, there is increasing evidence suggesting that autophagy serves a crucial role as a macrophage-intrinsic negative regulator of the inflammasome [46]. The stimulation of macrophages with an autoantigen-autoantibody immunocomplex leads to mitochondrial damage that further activates the inflammasome [46]. Given that autophagy and inflammasome activation are interrelated in autoimmune diseases [46, 47], the insights into the regulation of inflammasome activity by autophagy in RA should be investigated in future studies.

## Conclusions

The elevated autophagy expression with positive correlation to disease activity and inflammatory parameters in patients with RA suggests the involvement of activated autophagy in the pathogenesis of this disease. Our preliminary results also indicated that the therapeutic effectiveness of biologics may be related at least in part to their downregulation of autophagy expression. The elucidation of the pathogenic role of autophagy in RA may allow for the development of novel pharmaceutical agents in the future [44, 48].

## Additional file

Additional file 1: **Figure S1.** Representative cytometric histograms of Cyto-ID staining in circulating CD4^+^ T cells (A1), CD8^+^ T cells (A2), and CD19^+^ B cells (A3) from one patient with rheumatoid arthritis (RA) and one healthy control subject (HC). Comparisons of autophagosome levels reflected by Cyto-ID-staining MFI, in CD4^+^ T cells (B), CD8^+^ T cells (C) and CD19^+^ B cells (D) between patients with RA and HC. Data are presented as box plot diagrams, with the box encompassing the 25th percentile (lower bar) to the 75th percentile (upper bar). The horizontal line within the box indicates median value for each group. *p < 0.05 versus HC. Representative cytometric histograms of Cyto-ID staining in peripheral blood (PB)-derived granulocytes (E) and synovial fluid (SF)-derived granulocytes (F). Comparisons of autophagosome levels in PB-derived and SF-derived granulocytes in patients with RA (G). (TIF 1427 kb)

## Abbreviations

AA: Adjuvant-arthritis
ACPA: Anticitrullinated peptide antibodies
CRP: C-reactive protein
csDMARD: Conventional synthetic disease-modifying antirheumatic drug
Cyc: Cyclosporine
DAS28: 28-joint Disease Activity Score
ESR: Erythrocyte sedimentation rate
FITC: Fluorescein isothiocyanate
FLS: Fibroblast-like synoviocytes
HC: Healthy control subjects
HCQ: Hydroxychloroquine
IL-6: Interleukin 6
IL-6R: Interleukin 6 receptor
LC3: Light chain 3
mAb: Monoclonal antibody
MFI: Mean fluorescence intensity
MTX: Methotrexate
PBMC: Peripheral blood mononuclear cell
PC5: Phycoerythrin-cyanine 5
PerCP: Peridinin chlorophyll protein
RA: Rheumatoid arthritis
RF: Rheumatoid factor
RT: Room temperature
SF: Synovial fluid
SQSTM1: Sequestosome 1
SSZ: Sulfasalazine
TAC: Total antioxidant capacity
TNF: Tumor necrosis factor
