# Supplementary figures and images for: Association between autophagy and inflammation in patients with rheumatoid arthritis receiving biologic therapy
Source: Arthritis Res Ther. 2018 Dec 5;20:268. doi: 10.1186/s13075-018-1763-0 (PMC6280483; doi:10.1186/s13075-018-1763-0)

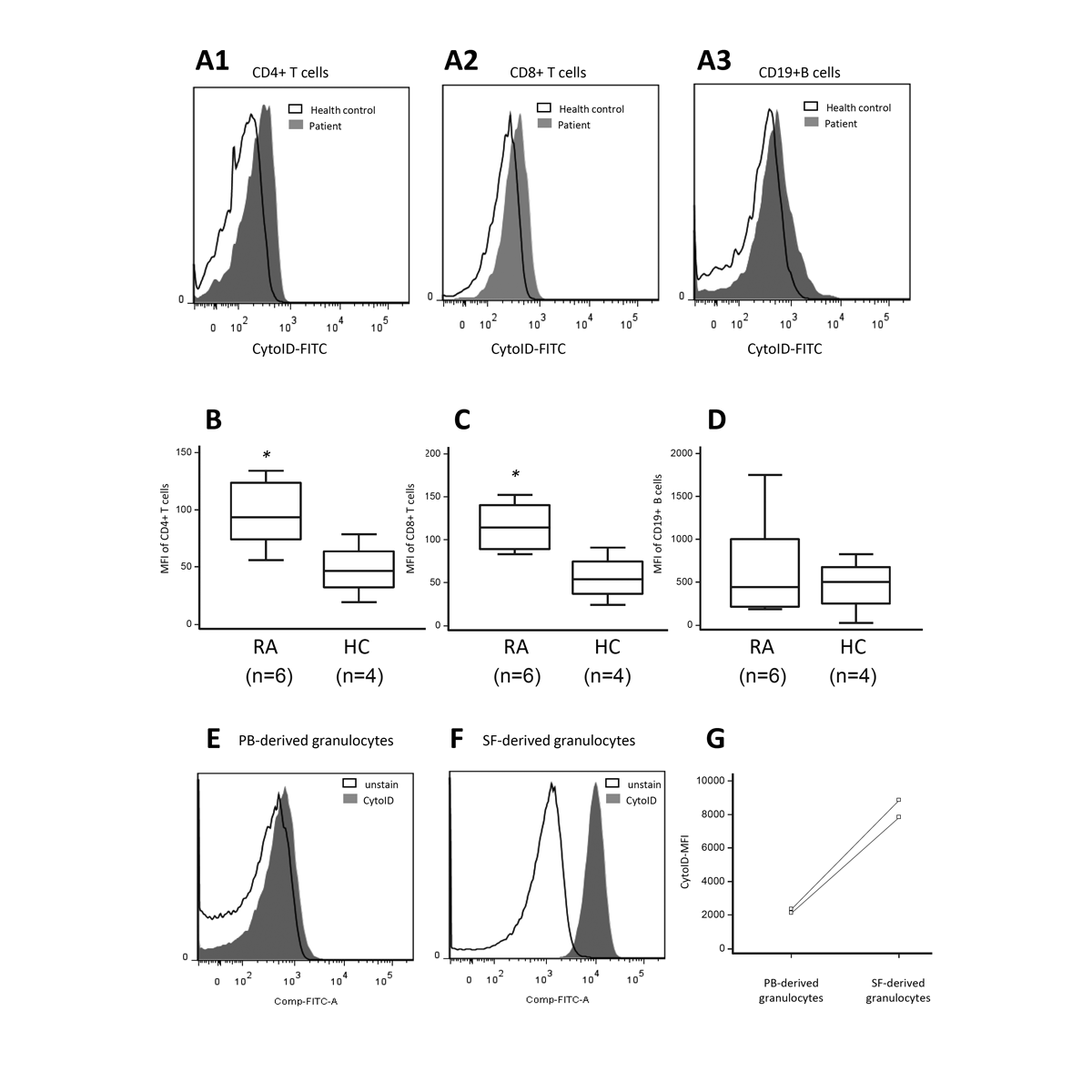

Supplement: Supplementary file 1 — Figure S1. Representative cytometric histograms of Cyto-ID staining in circulating CD4+ T cells (A1), CD8+ T cells (A2), and CD19+ B cells (A3) from one patient with rheumatoid arthritis (RA) and one healthy control subject (HC). Comparisons of autophagosome levels reflected by Cyto-ID-staining MFI, in CD4+ T cells (B), CD8+ T cells (C) and CD19+ B cells (D) between patients with RA and HC. Data are presented as box plot diagrams, with the box encompassing the 25th percentile (lower bar) to the 75th percentile (upper bar). The horizontal line within the box indicates median value for each group. *p < 0.05 versus HC. Representative cytometric histograms of Cyto-ID staining in peripheral blood (PB)-derived granulocytes (E) and synovial fluid (SF)-derived granulocytes (F). Comparisons of autophagosome levels in PB-derived and SF-derived granulocytes in patients with RA (G). (TIF 1427 kb) [file 13075_2018_1763_MOESM1_ESM.tif]
